# Do Medical Schools Need to Adapt Their Curriculum in Order to Teach Medical Students ‘Webside’ Manner? A Systematic Review

**DOI:** 10.1007/s40670-025-02498-2

**Published:** 2025-09-06

**Authors:** A. Newnham, T. Tattersall, J. Odendaal

**Affiliations:** 1 University Hospitals Coventry & Warwickshire, Coventry, CV2 2DX UK; 2 Division of Biomedical Sciences, Clinical Sciences Research Laboratories, Warwick Medical School, University of Warwick, Coventry, CV2 2DX UK

**Keywords:** Medical students, Virtual consultation, Telemedicine, Systematic review

## Abstract

**Background:**

Remote consulting was exponentially implemented secondary to the COVID-19 pandemic, and remains a staple of modern healthcare. Telemedicine consulting requires a different set of consultation skills collectively coined ‘webside manner’. Evidence suggests inadequate training is a barrier to effective teleconsulting. This review aims to systematically assess the effect of telemedicine consultation skills training for medical students.

**Methods:**

A systematic literature search was conducted using MEDLINE, PsycINFO, and EMBASE. Two independent reviewers screened articles from 1 January 2010 onwards. A mixed-methods approach was undertaken. Thematic analysis identified three reporting themes. Quantitative data was reported within these themes using descriptive statistics. Study quality was assessed using the MERSQI score.

**Findings:**

In total, 241 articles were obtained, 38 extracted for full text review, and 11 included. Three themes were identified: communication skills, doctor-patient relationship, and confidence in performing virtual consultations. Six out of seven studies reported improved communication skills following telemedicine training. Three studies report a positive impact on the doctor-patient relationship. Student confidence showed improvement in all reporting studies.

**Conclusion:**

This review demonstrates a positive association between telemedicine training and improved virtual consultation skills for medical students. The results are limited by the low quality and heterogeneity of included studies.

## Introduction

Telemedicine is a growing branch of modern healthcare practice. This reflects a combination of the increased demand placed on healthcare services and the need for accessible healthcare [1, 2]. Telemedicine encompasses a variety of methods for the provision of remote clinical services. The telephone-first initiative in primary care is a well-established public health initiative designed to reduce strain on primary care services [3]. In addition, the COVID-19 pandemic resulted in the rapid implementation of remote consulting and virtual platforms in both primary and secondary care [4]. Whilst the implementation of telemedicine was hastened due to COVID-19, both virtual consultations and telehealth now remain embedded in healthcare services globally [5, 6]. The increase in telemedicine’s popularity is reflected in the increased estimated global market value reaching $175 billion by 2026 [7]. The implementation of this consulting method into routine practice has seen doctors adapt their skills and develop new approaches to communication in a non-formalised fashion [8, 9]. Despite this, however, provision of training has varied amongst regions and countries. In addition, barriers to training access have been reported [10]. A 2024 survey of medical specialty educators examined training provisions before and after the COVID-19 pandemic. The results showed little to no training prior to COVID-19 but widespread implementation of locally led telemedicine training after the pandemic. This underscores the continued significance of telemedicine education [11]. Furthermore, the General Medical Council’s (GMC) Good Medical Practice guidance states doctors and medical students must ensure their knowledge and skills are up to date to avoid harm and to provide effective patient care [12]. Digital literacy and telemedicine consulting are skills future clinicians will need to acquire to maintain safe and effective care [13].

Training in telemedicine is integral to prepare clinicians for the projected future increase in remote clinical care [14]. In addition, when focussed on remote consultations, it has been demonstrated to improve confidence and self-efficacy [9, 15]. Two scoping reviews on the implementation of a telemedicine curriculum for student allied healthcare professionals highlighted positive responses to dedicated teaching on telemedicine skills [16, 17]. Concurrently, however, they reported a requirement for further research before widespread implementation can occur [16, 17]. No such review exists for medical students. Telemedicine poses challenges around access, acceptability, and ethics. In addition, it raises challenges to traditional methods of clinical assessment [18, 19]. One of the most frequently reported concerns is the impact remote consulting has on patient-clinician relationships [20]. This, in part, is due to the loss of non-verbal communication cues, particularly in the context of telephone consultations [8, 18]. Patients and clinicians recognise the importance of non-verbal communication on patient perceptions of their doctor and the role this plays in developing a rapport [21]. Capturing these non-verbal cues in remote consulting is the basis of the emergent webside manner, a term analogous to the traditional bedside manner [22, 23].

Lack of formal training has been demonstrated to be both a barrier to telemedicine implementation and a concern to doctors in training [10, 14]. A mixed-methods study of 100 General Practitioner (GP) trainees highlighted a lack of confidence and training in the skills required to undertake remote consultations [15]. Indeed, this has been recognised by the National Institute for Health Research, who have identified the need to develop new and evolving approaches to healthcare training with increased digitalisation [24]. Given the lack of confidence and training in telemedicine delivery, we would suggest that medical school may represent an opportune time to address telemedicine education. Medical students have been exposed to virtual consulting due to COVID-19, with many undertaking virtual consultations as part of their training [4, 25]. Furthermore, virtual consulting is commonplace in GP placements, which are an integral component of medical education. Therefore, the focus of this review is telemedicine training for medical students [15].

This review aims to systematically review the literature on the provision and perceived outcomes of telemedicine consultation and communication skills training for medical students. We opine that early telemedicine training as part of the medical school curriculum may improve the confidence and competence of future clinicians.

## Methods

A systematic review of published studies between 1 Jan 2010 and 9 Nov 2023 was performed using keywords related to telemedicine, communication, and medical education. Articles prior to 2010 were excluded to ensure the literature remained consistent with technological advancement within healthcare within the last decade. Although telephone consultations have been present in primary care before 2010, rates of usage were low, only exceeding 10% as late as 2008/2009 [26]. Therefore, 2010 was considered appropriate to capture early telephone consulting studies.

MEDLINE, PsycINFO, and EMBASE databases were searched using the following key search terms and related terms: ‘remote consultations’, ‘medical education’, and ‘simulation training’. We used Boolean operators to get more precise and relevant search results. Searches were refined to remove duplicates. For further detailed search terms, see Appendix 1.

Two authors independently undertook primary and secondary screening of the articles retrieved in the search against the inclusion criteria. Any conflicts were resolved through consensus. To be suitable for inclusion, the following criteria had to be met: participants were medical students, the intervention was a telemedicine curriculum or course, and the primary outcome measure was reporting of effect upon communication skills. Articles were excluded if they were not available in the English language, or if they were opinion pieces, conference abstracts, and letters. Only remote consultations in the form of video or telephone call were included, whilst other telehealth concepts, for example, e-consults, were excluded. This is due to a different set of communicative challenges with approaches where no direct patient contact is required; these include e-consulting, advice and guidance, and chatbot healthcare.

The included studies consisted of mixed methodologies. To provide structure and guide data collection, the use of emergent theme thematic analysis was employed to avoid relying on prior assumptions [27]. The included studies were reviewed multiple times by the primary author to promote familiarity with the data. The texts were then coded to identify themes relevant to the research question. Thematic analysis was concluded at the point of thematic saturation, resulting in emergent themes. Deeper thematic analysis was not possible due to limited reporting of primary qualitative data within the individual studies. Most of the primary studies presented only the authors’ interpretations and discussions of their qualitative findings. As a result, the emergent theme analysis synthesised these interpretations to enrich the data, leading to the themes presented in this review. Three emergent themes were agreed upon by all authors: communication skills, doctor-patient relationship, and confidence in performing virtual consultations. Digital literacy, whilst an important part of virtual consulting, was excluded as it did not address the research questions primary outcome. The primary author is trained at master’s level in qualitative research methodology and mixed-methods approaches, whilst the senior author has previously published qualitative meta-syntheses.

The quantitative results are displayed within these three themes. Data was extracted by the primary author and checked by a second author. Quantitative data is reported as simple descriptive statistics comparing numerical values where applicable and questionnaire variables, often Likert responses.

A quality assessment was undertaken by two authors independently using the Medical Education Research Study Quality Instrument (MERSQI), a specific tool for medical education research [28]. Differences of opinion were resolved by discussion and consensus. In all cases, consensus agreement was reached.

## Results

**Fig. 1:**
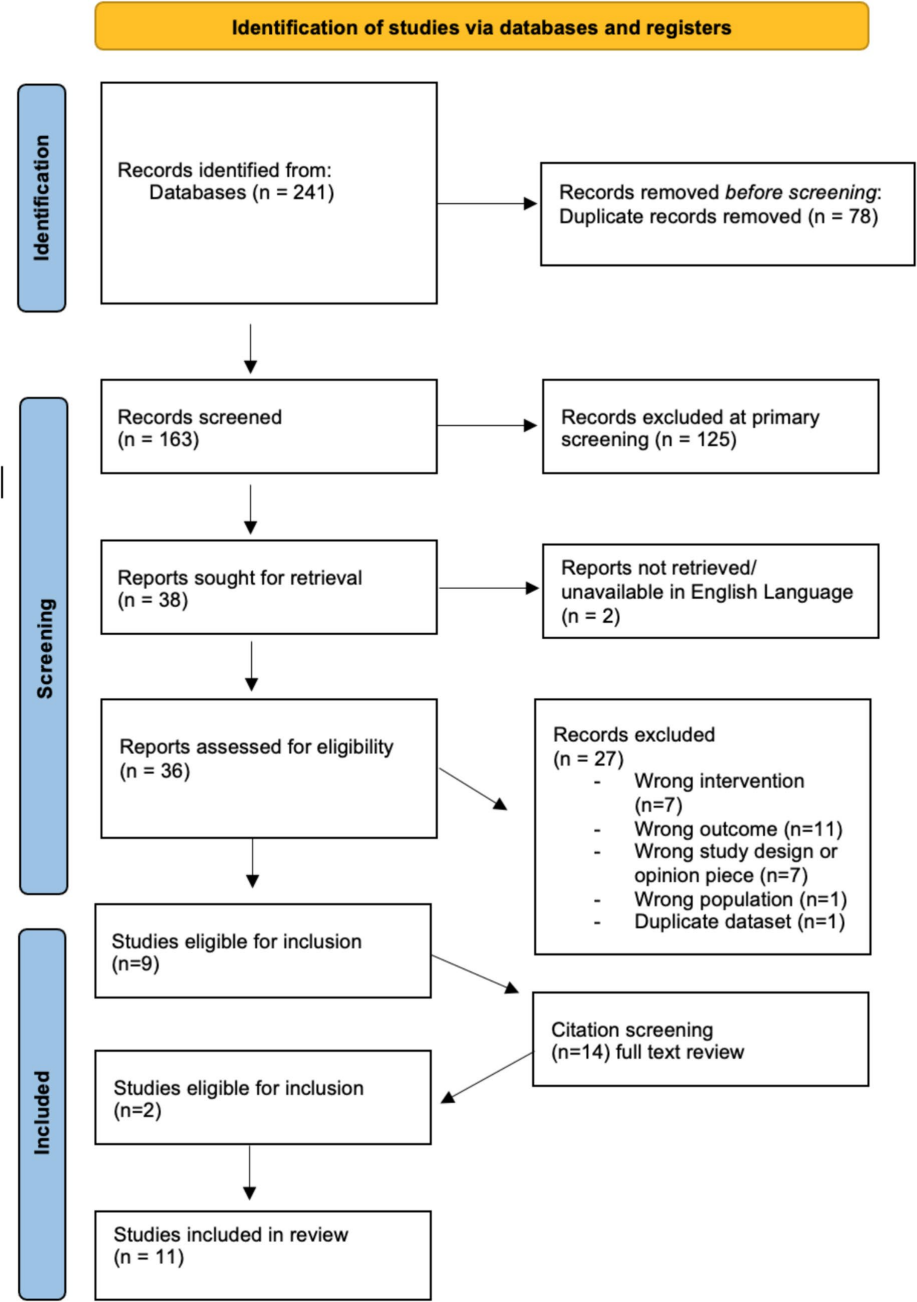
PRISMA diagram

Figure 1 demonstrates the PRISMA diagram for article selection. The primary search produced 241 citations. After removal of duplicates and review of abstracts, 38 full-text articles were extracted for review. Of these, 27 were excluded, with a total of 11 articles meeting inclusion criteria. The 11 articles were assessed for quality using the MERSQI scoring system. The MERSQI looks at 6 domains: study design, sampling, data type, validity of evaluation tool, data analysis, and outcome, producing an overall score [28]. The maximum score is 18, with higher scores correlating to better quality studies. Scores for the studies ranged from 6 to 12, with a median score of 8.2/18. For individual study assessments, see Appendix 2. Overall, the quality of the included studies is low, as demonstrated by the quality assessment, with average publishable scores being 10.7, with a cut-off of 9.0. Data provision is lacking in several of the studies, particularly in those that demonstrate a qualitative element to their study. Methodology and analysis reports are limited. This may reflect the rapidly evolving nature of COVID-19 and the demand for ‘quick’ research results to guide a change in practice.

### Study Characteristics

The 11 studies include a total of 809 medical students ranging from first year to final year students. Over half the studies included were from the Americas; 5 out of 11 from the USA and 1 out of 11 from Canada. The remaining 5 studies were undertaken in the UK, Australia, and Germany. The sample sizes varied significantly ranging from 5 students to 153 students. Nearly all the studies used students’ perceptions and opinions of their own personal skills as an evaluation measure, with only 2 out of 11 studies using objective data provided by the simulated patient. Nine out of 11 use self-reported participant questionnaires only. One out of 11 studies uses a combination of objective and self-reported participant feedback for data collection. The loss to follow-up also varied greatly from 0% loss to follow-up through to 55%. Table 1 shows the characteristics of the included studies and the themes that they apply to.

**Table 1:** Study characteristics of included articles [29–39]

Author (date) [Country]	Participant details	Evaluation tool	Outcome reporting	Survey completion rate	Theme	MERSQI score
Afonso (2019) [USA]	1 st year medical students (*n* = 122)	Self-reported post-intervention questionnaire Qualitative data from open ended questions	Likert scale with percentages 5 quotes	47% N/A	Communication skills	6
Bramstedt (2014) [Australia]	2nd year medical students (*n* = 4), with 2nd year medical student observers (*n* = 23)	Two question narrative feedback (qualitative data) Observers—self-reported post-intervention questionnaire—Likert scales and Yes/No	Few quotes Likert scales and Yes/No questions	(1) 100% (2) 65%	Communication skills Doctor-patient relationship	8.5
Booth (2022) [Scotland, UK]	5th (final) year medical students (*n* = 21)	Pre- and post-intervention self-reported questionnaire Online survey at 4 weeks	Likert scales with numerical valued applied for mean, mean difference, and standard deviation Percentages	95% 80%	Overall confidence in virtual consulting	7.5
Gunner (2021) [UK]	Final year medical students (*n* = 40)	Pre- and post-intervention self-reported questionnaire	Likert scales with numerical valued applied for mean, mean difference and standard deviation	85%	Overall confidence in virtual consulting	7.5
Kumra (2022) [USA]	2nd year medical students (*n* = 120)	Post-intervention simulated patient feedback Self-reported post-intervention questionnaire	Numerical score provided Likert scales with percentages	100% 88%	Communication skills Overall confidence in virtual consulting	10.5
Mulcare (2020) [USA]	Clinical medical students, year unreported (*n* = 98)	Pre- and post-intervention self-reported questionnaire Qualitative data not relevant to primary outcome of this review	Likert scales with percentages	100%	Communication skills	7
Murphy (2023) [USA]	2nd year medical students (*n* = 60)	Pre- and post-performance feedback by the simulated patient	Median scores with IQR and median score difference with IQR	95%	Communication skills	7.5
Newcomb (2021) [Canada]	4th year medical students (*n* = 5)	Pre- and post-intervention self-reported questionnaire Qualitative feedback via open questions	Likert scales 3 quotes provided	100%	Doctor-patient relationship	12
Rienitis (2015) [Australia]	3rd year graduate entry medical students (*n* = 71)	Pre- and post-intervention self-reported questionnaire	Likert scales with percentages	83%	Overall confidence in virtual consulting	7.5
Vogt (2022) [Germany]	Preclinical medical students, year not reported (*n* = 92)	Self-reported post-intervention questionnaire for simulated practice Pre- and post-intervention self-reported questionnaire	Likert scales with numerical valued applied for mean, mean difference and standard deviation	45% Not reported for pre and post	Communication skills Doctor-patient relationship	8.5
Walker (2019) [USA]	2nd year medical students (*n* = 153)	Pre- and post-intervention self-reported questionnaire	Likert scales with numerical valued applied for mean, mean difference and standard deviation	60%	Communication skills Overall confidence in virtual consulting	8

### Methods of Training or Curriculum Implementation

Curriculum design and training delivery varied considerably across the 11 studies. Although all included some form of simulated practice, there were notable differences in theoretical content, training duration, delivery methods, and the nature of pre-intervention learning or information provided. The specific approach taken in each study is outlined in Table 2.

**Table 2:** Intervention characteristics of included studies [29–39]

Author (date)	Pre-intervention learning	Facilitated intervention	Length of facilitated training
Afonso (2019)	Step by step written guides and examination videos	Faculty demonstration Simulated patient practice Case-based discussion	2 hr
Bramstedt (2014)	None	Simulated patient practice with a lived experience patient. Questions students asked were pre-prepared by faculty	1 hr
Booth (2022)	None	Two sessions: 1. Lecture for theoretical provision, simulated practice consultations and group debrief 2. Simulated practice consultations and group debrief	2–3 hr
Gunner (2021)	None	Lecture for theoretical provision—‘Introduction to video consultation’ Two stations (originally three in pilot): 1. Technology set up, observe two example video consultations, and simulated practice. 2. Patient selection and ethical issues—discussion of mock cases, suitability for video, and ethical considerations with video consultations	2 hr
Kumra (2022)	Handout provided to read Case completion on an electronic record	Simulated practice under examination conditions	15 mins
Mulcare (2020)	‘Homework exercise’—submitted a self-recording of themselves delivering instructions to a patient regarding a soft tissue injury	Lecture for theoretical provision Debrief session ‘Homework exercise’ Table top exercises and simulated practice covering four areas: on-camera etiquette, verbal and non-verbal communication skills, physical examination skills and appropriate disposition planning to ensure patient safety	8 hr
Murphy (2023)	Pre-intervention simulated patient practice encounter	Group 1—Simulated roleplay session Group 2—Faculty-led demonstration	Not reported
Newcomb (2021)	None	Lecture for theoretical provision Discussion of student experiences Simulated roleplay practice Group reflection on simulated roleplay practice	2 hr
Rienitis (2015)	None	Four stations: 1. ‘Establishing connections’—practical skills of using technical equipment 2. ‘Role-playing a teleconsultation’—student role play patient and doctor 3. ‘Viewing a teleconsultation’—observation of a filmed example 4. ‘Discussing the ethics and dilemmas’	2 hr
Vogt (2022)	Online resources available including: handover training, example cases	Lecture for theoretical provision on communication, error management and patient safety Simulated patient practice in small groups Simulated handover practice	Not reported
Walker (2019)	None	Lecture for theoretical provision Observation of example consultations Simulated practice	1 hr

### Effectiveness of Training or Curriculum

Direct comparison of the included studies was difficult due to significant heterogeneity in methodological approaches, intervention implementation, and outcome reporting as seen in Table 2. As a result, narrative synthesis has been undertaken. Thematic analysis of included studies identified three focussed themes for reporting data: confidence in performing virtual consultations, communication skills, and establishing the doctor-patient relationship or rapport. Digital literacy is not reported as this does not address webside manner. Finally, participants’ overall perception of the interventions provided was included. This is deemed relevant as an intervention needs to be acceptable and appropriate for its user and provide stakeholder feedback for future researchers.

### Communication Skills

Seven out of 11 studies include questions directly evaluating communication skills in their study outcomes. The majority demonstrate positive associations as a result of their intervention, with only one demonstrating no change following the telemedicine training (Table 3). Mulcare et al. is not shown in Table 3, as they provide no numerical or descriptive data but simply provide a quote that ‘almost all learners concluded that the simulation session would alter their approach to communicating with patients over a virtual medium going forward’ [34].

**Table 3:** Results of intervention on communication skills [29, 30, 33, 35, 38, 39]

Study	Pre-intervention	Post-intervention	Difference	Positive/negative/indifferent association
**Afonso**		53/57 (93%) strongly agreed or agreed their communication skills improved		Positive
**Bramstedt**		12/15 (80%) strongly agreed or agreed their communication skills improved		Positive
**Kumra**		93% participants score 76–100% on communication skills		Positive
**Murphy**	8	8	0	Indifferent
**Vogt** Communication with patient Inter-professional communication	Mean 3.83	Mean 4.59	Mean difference 1.10 [SD 1.25]	Positive
	Mean 3.08	Mean 4.59	Mean difference 1.51 [SD 1.45]	Positive
**Walker**^a^	Mean 2.32	Mean 4.25	Mean difference 1.93	Positive

^a^Walker report on non-verbal communication only; this score reflects this

Whilst the majority of studies rely on self-reported improved communication skills, Kumra et al. provide a numerical score calculated by simulated patient feedback [33]. However, it is not clear how this score is impacted by the session, as a pre-intervention score is not performed. This is similarly seen for Afonso and Bramstedt et al. [30]. Furthermore, it is not entirely clear how this score is generated, despite providing the simulated patient checklist in the appendices.

Of the 11 studies, only Walker et al. specifically reported on body language when performing a virtual consultation [39]. They demonstrated a positive association with training [39]. Body language is reported as one of the most important differences between face-to-face and virtual consulting in the literature; therefore, it may be a consideration for future research within telemedicine training provision [40]. Although most studies reported overwhelmingly positive effects on communication skills for virtual consulting following their training interventions, significant limitations in study design and reporting mean that these findings should be interpreted with caution.

### Doctor-Patient Relationship

Three out of 11 studies specifically evaluated the intervention effect on empathy and establishing a doctor-patient relationship. Vogt et al. evaluated student confidence on building a doctor-patient relationship during a virtual consultation with a mean score of 4.05 (SD 1.12) [38]. This variable was not evaluated pre-intervention; therefore, there is no mean difference limiting the assessment of intervention efficacy. Similarly, Bramstedt et al. evaluated empathy towards patients in the virtual setting [30]. They report 12 out of 15 participants strongly agreed or agreed that the session improved their empathy towards patients in this setting, equating to 80% [30]. They also report all 15 participants strongly agree or agree that the session has aided in professionalism and behaviour training [30]. Newcomb et al. are the only study that evaluates empathy pre- and post-session using Likert [36]. Prior to the session, the majority of students felt fairly confident in demonstrating empathy; however, following the session, the majority felt completely confident demonstrating a change post-intervention, with exact numbers not provided [36]. Whilst these 3 studies demonstrate a positive association with telemedicine training and confident provision of the doctor-patient relationship, the numbers are small (*n* = 101) and therefore the significance is unestablished, limiting interpretation.

### Confidence in Performing Virtual Consultations

Whilst specific reporting on non-technical skills such as developing empathy and establishing a rapport is not present, studies did report on general consulting and history taking, a key part of webside manner. A significant part of history taking includes effective communication with the patient. Therefore, overall confidence in performing virtual consultations, including history taking, was evident within the review. This applies to 5 out of 11 studies.

Booth et al. and Gunner et al. both applied numerical values to Likert scales, calculating mean confidence scores pre- and post-intervention [31, 32]. Both present improvement in confidence following the telemedicine training sessions. Booth et al. evaluate mean confidence in history taking increases from 3.4 to 4.45 post-intervention, with a mean difference of 1.05 [31], whilst Gunner et al. evaluated in video consultation skills, with perceived student confidence increasing from 2.32 (SD 0.83) pre-intervention to 3.97 (SD 0.38) post-intervention [32]. This represents a significant mean difference (*p* < 0.01)[32]. Similarly, Walker et al. report a pre-intervention mean score of 2.73 and post-intervention score of 3.62 (mean difference 0.89) when evaluating student comfort conducting telemedicine encounters[39]. Kumra et al. support this, stating 93% of students post session strongly agreed or agreed that the session increased their confidence in history taking in the virtual setting [33]. Rienitis et al. further support this by evaluating student confidence in conducting the complete virtual consultation [37]. Prior to the intervention, 6 students provided positive responses equating to 10.2%, increasing to 41 post session, equating to 69.5% [37]. These studies demonstrate a positive association with telemedicine training and self-reported confidence in virtual consulting; however, the quality of evidence is low and should be interpreted with caution.

### Overall Value of the Session

The value of the session was reported quantitatively as percentages in 4 out of 11 studies with Booth et al., Vogt et al., and Mulcare et al. presenting positive reactions to the training provided [31, 34, 38]. Ninety-seven percent of students felt the course was useful in Mulcare et al., 83.4% of students rated the course as very good or good in Vogt et al., and 87.5% found the session very useful in Booth et al., with none reporting the session as not useful at all [31, 34, 38]. However, Rienitis et al. report that only 45.8% felt the course was valuable to their learning meaning the majority felt the course was not valuable [37]. Other studies included quotes from students related to the course set up and value. However, they did not present any qualitative methodology or evaluation and therefore these were not included in this review due to limited additional value added and an inability to assess trustworthiness of this data. Whilst overall value of the session does not answer the question posed by this review, it is an important consideration when considering any intervention or curricular change. It has to be acceptable to students and be perceived as important to encourage engagement and change [16].

## Discussion

Telemedicine was exponentially implemented due to the COVID-19 pandemic and remains a pertinent approach to modern healthcare worldwide [4]. The GMC has made it evident that graduating doctors need to have safe and effective telemedicine skills; however, medical school curriculums have yet to incorporate training as standard in many countries across Europe [10, 11, 25]. In this study, we aimed to review the telemedicine curriculum with a focus on ‘webside manner’ encompassing communication skills as the main focus of training. This review did not assess data surrounding digital literacy as it was assumed that basic digital literacy is present amongst the majority of medical students. We recognise, however, the importance of digital literacy in providing all aspects of modern healthcare including teleconsulting.

We found a limited number of studies with varied approaches to telemedicine curricular design and implementation for medical students. We have demonstrated that there are a variety of approaches that can be adopted in order to provide a telemedicine curriculum to medical students. There is not enough evidence to suggest a superior approach at present. Furthermore, the literature thus far does not allow us to make recommendations on how or when to implement telemedicine education into the medical school curriculum.

Additionally, studies suffered from significant heterogeneity of design and outcome impacting interpretation. Studies also were of low quality with a high risk of bias impacting the ability to infer the efficacy of telemedicine training on improving communication skills and webside manner. The majority of studies rely on self-reporting for data collection. A significant number of the included studies used only post-intervention outcomes measures, resulting in an inability to assess knowledge acquisition effectively. Whilst self-reporting offers valuable insights into students’ self-perceptions, incorporating additional evaluation methods could enhance the validity and overall quality of the research. Future research would benefit from rigorous methodology to assess knowledge acquisition [41]. Furthermore, a move from self-evaluation would be beneficial in guiding best practice.

What is known is that telemedicine consulting requires a different set of skills, and currently, training is lacking for qualified medical professionals, let alone medical students [15, 40]. Two scoping reviews of allied healthcare professionals support the positive association between telehealth training and competency demonstrated in this review [16, 17]. Further research is required, including the need for a qualitative approach to the research question, to ensure stakeholders such as faculty and medical students’ opinions, needs, and barriers to adoption are recognised.

This review has demonstrated that most of the included studies reported positive associations with training, including improved confidence in virtual consulting, improved communication skills, and beneficial effects on empathy and establishing doctor-patient relationships in the virtual setting. Furthermore, 3 out of 4 studies report the curriculum intervention to be valuable to students, inferring that telemedicine skills training would be acceptable to students if included in medical school curricula in the future [31, 34, 38]. Further research is required to generate recommendations on how and when to implement this.

### Strengths and Limitations of the Present Study

This review is necessary to provide information to medical educationalists to ensure future clinicians provide safe and effective teleconsultations. We include up-to-date citations encompassing pre-, during, and post-COVID-19 literature correlating with the increased implementation of telemedicine into the health service during these time periods.

The primary limitations of this review are the exclusion of articles that were not available in the English language and the inclusion of low-quality studies. The included studies were inconsistent in their interventional approaches, evaluation methodology, sample sizes, and settings. Furthermore, the average quality score of 8.2 on MERSQI is below the average accepted 10.7 and threshold of 9.0 for publication [40]. This reflects the relative novelty of the subject and rapid implementation and reporting secondary to the COVID-19 pandemic. As a result, it poses significant challenges with data analysis due to lack of comparability. A systematic approach to data extraction and inclusions reduces this risk of bias. However, data synthesis has an element of subjectivity due to the nature of the topic and differing study characteristics. In order to minimise the effect of author interpretation, two authors independently reviewed emerging themes, and consensus was reached.

## Conclusion

This review has demonstrated that telemedicine training for students positively affects student confidence in virtual remote consulting. It has demonstrated that perceived communication skills improve with telemedicine training and the ability to establish the doctor-patient relationship virtually also improved following training. Despite this, the methodology of telemedicine curriculum development, timing of implementation, and feasibility of implementation into medical school curricula require further research before recommendations can be made. Evident from this review is the significant heterogeneity in studies approaches thus far. A multicentre standardised approach would be beneficial to confirm the effectiveness of telemedicine training upon communication skills and readiness to practice virtual consulting. It is clear that telemedicine will continue to persist in healthcare, and there is an urgent need to support the provision of effective training to support this demand [5, 9].

## Data Availability

No original data is included within this review, all data extracted is publically available within the studies.

## Appendix 1 Medline search

A primary search was conducted using the following search terms:

[“Telemedicine” OR “Telehealth” OR “Virtual Consultation” OR “Remote Consultation” or “Virtual Care”] AND [“Education, Medical” OR “Education, Medical, Undergraduate” OR “Medical Students” OR “Medical Schools”] AND [“Training” or “Communication Skills” or “Simulation”] in 2020. This search is repeated in 2023 capturing post-COVID studies. No limits were applied.

Embase had a limit of no Medline records, as did PsycINFO with the same search terms as above.

## Appendix 2

See Table 4.

**Table 4:** MERSQI Scores for included studies–average between two independent authors

Author	Study design	Sampling (institution/response rate)	Data type	Validity of evaluation instrument	Data analysis (sophistication/appropriateness)
Afonso (2019)	1	1	1	0	2
Bramstedt (2014)	1	1.5	3	0	2
Booth (2022)	1.5	2	1	0	2
Vogl (2022)	1.5	1	1	0	3
Gunner (2021)	1.5	2	1	2	3
Kumra (2022)	1	2	1	0	2
Mulcare (2020)	1.5	2	1	0	2
Murphy (2023)	2	2	3	1	3
Newcomb (2021)	1.5	2	1	0	2
Rienitis (2015)	1.5	2	1	0	3
Walker (2019)	1.5	2	1	1	2

## Declarations

No ethical approval was required for this study as no primary data was collected,; all data was already available and published with appropriate consent via authors of primary publications.
